# Causal Judgment in the Wild: Evidence from the 2020 U.S. Presidential Election

**DOI:** 10.1111/cogs.13101

**Published:** 2022-02-05

**Authors:** Tadeg Quillien, Michael Barlev

**Affiliations:** ^1^ School of Informatics University of Edinburgh; ^2^ Department of Psychology Arizona State University

**Keywords:** Causality, Causal judgment, Causal selection, Counterfactuals, Computational modeling

## Abstract

When explaining why an event occurred, people intuitively highlight some causes while ignoring others. How do people decide which causes to select? Models of causal judgment have been evaluated in simple and controlled laboratory experiments, but they have yet to be tested in a complex real‐world setting. Here, we provide such a test, in the context of the 2020 U.S. presidential election. Across tens of thousands of simulations of possible election outcomes, we computed, for each state, an adjusted measure of the correlation between a Biden victory in that state and a Biden election victory. These effect size measures accurately predicted the extent to which U.S. participants (*N* = 207, preregistered) viewed victory in a given state as having caused Biden to win the presidency. Our findings support the theory that people intuitively select as causes of an outcome the factors with the largest standardized causal effect on that outcome across possible counterfactual worlds.

## 1 Introduction

Imagine that scientists designed a machine that gives complete explanations for why things happen. Upon its unveiling, this machine is tasked with explaining why a forest caught on fire. After hours of churning data, the machine begins: lightning struck a dead tree, and this tree was surrounded by dry leaves after months of drought, and the surrounding atmosphere contained 21% oxygen, and the laws of thermodynamics postulate that… and so on it goes. A human, in contrast, might simply say “A lightning bolt caused the forest fire.”

When explaining why an event occurred, people intuitively highlight some causes while ignoring others. How do people decide which causes to select? Researchers have suggested that people compute an index of the “actual causal strength” of various factors that lead to an event, and that they consider the factor(s) with the highest causal strength as “the” cause(s) of the event (e.g., Morris et al., 2018; Icard et al., 2017; Gerstenberg et al., 2021; Quillien, 2020).

But how do people compute this index of causal strength? When scientists need to quantify the strength of an effect, they use statistical measures of “effect size.” For instance, to compute the causal effect of exercise on heart rate, we could randomly assign a group of participants to levels of exercise ranging from very mild to very strenuous, measure their heart rate, and then calculate the Pearson correlation between the two variables.

At first sight, this effect size approach cannot be used to estimate the causal strengths of factors leading up to a single event, as it is mathematically impossible to compute an effect size for a single observation. According to a recent proposal, however, the mind achieves this seemingly impossible feat by considering counterfactual worlds – alternative ways in which the event could have happened (Quillien, 2020). For instance, when making a judgment about the cause of a forest fire, people may consider many counterfactuals of that event. Across these counterfactuals, the correlation between lightning striking a tree and fire is high (in most possible worlds where lightning strikes a tree, there is fire, and there are relatively few possible worlds where there is fire but lightning did not strike a tree); by contrast, the correlation between oxygen in the air and fire is low (there are no possible worlds where there is fire without oxygen in the air, but there are very many possible worlds where there is oxygen in the air without fire). Therefore, we judge the lightning bolt, and not the oxygen, to be the cause of the fire.

The proposal that judgments of causation rely on counterfactual thinking has a long history in philosophy (Hume, 1748; Lewis, 1973; Hitchcock & Knobe, 2009), law (Hart & Honore, 1985), and psychology (Kahneman & Miller, 1986; Gerstenberg et al., 2017; Kominsky & Phillips, 2019; Henne et al., 2019, 2021b). Yet, that our minds consider counterfactuals in this way may feel far‐fetched. We are not, after all, consciously aware of doing so most of the time. But this proposal is bolstered by empirical findings from a separate line of research, increasingly popular in cognitive science, on how people use *probabilistic causal models* to predict the future (Griffiths et al., 2010; Tenenbaum et al., 2011).

Consider physical reasoning. When tracking a moving object, the mind may use an internal physics simulator – akin to a game physics engine – to compute the probability of different possible future trajectories for that object (Battaglia et al., 2013; Ullman et al., 2017).^1^ The physics engine is a *causal* model, because it represents the world in terms of causal laws (e.g., some approximation of Newtonian mechanics) regulating a set of entities (e.g., billiard balls). It is also a *probabilistic* model, because it incorporates the fact that our knowledge and/or predictions are uncertain.^2^

Causal models are not only useful for predictions: they can also be used to reason about what could have been (Pearl, 2000; Lucas & Kemp, 2015). For example, the same physical laws used to predict the trajectory of a billiard ball can be used, after the fact, to estimate the probability that the ball could have missed the pocket instead of entering it. Crucially, empirical evidence suggests that people reason about counterfactuals in this way (Rips, 2010; Lucas & Kemp, 2015).

It is natural to assume that when people make causal judgments about an event, they generate counterfactuals by using the probabilistic causal model they would have used to make predictions about the event (see Icard et al., 2017). People do not tend to generate counterfactual situations that are very unlikely according to their probabilistic causal model–they rarely look at a fire and think “what if there had been no oxygen in the air to fuel the combustion?” (Kahneman & Miller, 1986; Lucas & Kemp, 2015; Byrne, 2016). Put more formally, one can assume that people tend to generate counterfactuals in proportion to their prior probability.

In sum, people may make causal judgments by (1) generating counterfactuals to an event in proportion to their prior probability,^3^ and (2) across these counterfactuals, compute a measure of effect size between different factors and the outcome. Here, we call this model the counterfactual effect size model (CESM; Quillien, 2020).

The CESM parsimoniously explains many human causal intuitions. Notably, it gives a natural explanation to a complex phenomenon that has to do with the way that prior probability influences causal judgment (Icard et al., 2017; Gerstenberg & Icard, 2020). Consider Bob, who graduated after passing both his history and mathematics examinations. History is a very easy subject for Bob, but he usually struggles in mathematics. If the scenario specifies that Bob needed to pass both classes in order to graduate, people tend to say that he graduated *because he passed mathematics* (the unexpected event). But if the scenario specifies that Bob needed to pass at least one of the examinations, people tend to say that he graduated *because he passed history* (the expected event).

In more formal terms, when two factors lead to an outcome, and both factors were necessary for the outcome, people say that the factor that was a priori the least likely was the cause. But this effect reverses when either factor would have been sufficient to produce the outcome. This pattern of effects has been replicated many times across a large range of stimuli (Icard et al., 2017; Gerstenberg & Icard, 2020; Kominsky & Phillips, 2019; Quillien & German, 2021; O'Neill et al., 2021; Kirfel et al., in press; see also Henne et al., 2019, 2021b).

The CESM gives a simple explanation to this finding. In the scenario where Bob needed to pass both examinations to graduate, Bob's successful graduation is most highly correlated (across counterfactuals) to his passing mathematics.^4^ But in the scenario where Bob needed to pass at least one examination, Bob's graduation is most highly correlated with whether he passed history (see Icard et al., 2017 for an alternative interpretation).^5^

Additionally, the CESM is able to reproduce human causal judgments in experiments manipulating the prior probability of events in a fine‐grained way (Morris et al., 2019; O'Neill et al., 2021). Indeed, it reproduces subtle nonlinear patterns in people's judgments, and has a significantly closer fit to these data than other prominent models.

However, data supporting the CESM come from experiments where participants had to reason about very simple causal structures. For instance, participants were asked about a simple casino game (Morris et al., 2019) or a collision between a few billiard balls (Gerstenberg & Icard, 2020; Henne et al., 2021a). But can the CESM explain causal judgments in the real world, where events have a multitude of causes?

Here, we report a test of the CESM “in the wild,” using the case study of the 2020 U.S. presidential election. We asked a sample of U.S. participants to, for each state in which Biden won the popular vote, rate the extent to which winning in that state caused Biden to win the presidency. We then compared their judgments to the CESM's predictions.

The CESM assumes that people have a mental representation of the causal structure of the relevant domain, which includes its causal laws and the prior probabilities of different factors. With regard to the U.S. presidential election, most Americans know the rules: for instance, candidates win electoral votes by winning the majority in a state, and the candidate with the most electoral votes is elected president. Additionally, most Americans have intuitions about the prior probabilities of different factors: for example, a Democratic victory is almost certain in California but not in Florida.

We use election forecasts as a proxy for people's mental representation of the causal structure of the election. Assuming that both laypeople and election forecasts approximately track the same ground truth about U.S. politics, election forecasts can be used as a stand‐in for lay representations. Election forecasts derive their predictions by simulating tens of thousands of possible election outcomes, in proportion to their estimated probability. We can directly use these simulations to compute the effect size measures defined by the CESM.

In addition to the CESM, we test whether two other existing formal models of causal judgment (preregistered) and three other models of general causal strength (exploratory) can explain human causal intuitions about the election. We use election forecasts to derive predictions for all but one of these alternative models. Our use of forecasts as a stand‐in for lay mental representations does not, therefore, advantage any model in particular.

## 2 Method

### 2.1 Behavioral study

#### 2.1.1 Participants

Data collection took place on November 29, 2020, which was about a month after the presidential election (November 3), after Biden had formally declared his victory (November 7), and about when major news media were reporting on challenges to the election results in specific states as largely resolved.

U.S. participants (*N* = 207 after exclusion; 51% female; mean age = 33) recruited using Prolific completed a brief survey in exchange for monetary compensation. Participants were excluded if they failed attention and comprehension checks. Additionally, because it was important for this study that participants be politically involved and knowledgeable, participants were also excluded for reporting to not have closely followed the U.S. 2020 presidential election or to have failed two or more questions on a basic five‐question multiple‐choice quiz of U.S. political knowledge (modified from Delli Carpini & Keeter, 1993; see Supplementary Information for full quiz). Twenty three percent of participants were excluded for not being politically involved or knowledgeable enough. The sample size and exclusion criteria were preregistered. See preregistration at https://osf.io/m3hgf for more details. The sample of participants was skewed toward liberal, with 60% of participants identifying as Democrat, 22% as Independent, 10% as Republican, and 8% as “Other.” Additionally, on a 1 = Very Conservative to 7 = Very Liberal scale, the average response was 5.44 (between 5 = Slightly Liberal and 6 = Moderately Liberal).

#### 2.1.2 Materials and procedure

Participants were shown a map of the U.S. 2020 presidential election results, with states Biden won highlighted in blue and states Biden lost highlighted in red. Within each state was displayed the abbreviation for that state (e.g., CA for California); there was no information displayed about the number of electoral votes per state. With this map on the screen, participants were asked, for each state Biden won, how much they agreed with the statement “Biden won the presidency because he won [state].” (from 0 = do not agree at all to 10 = agree very strongly). The states were displayed one at a time and in randomized order. The survey was administered via Qualtrics.

### 2.2 Computational modeling

We preregistered predictions for three different computational models of human causal judgment. After data were collected and analyzed, we decided to explore the predictions of three additional computational models. We derive the actual causal strength, as predicted by each of these models, of the event “Biden wins state S” for each state Biden won. For readability, we placed many of the technical details in the Supplementary Information, available at https://osf.io/6jgez/.

We note that these models are general enough that we did not have to make specific adjustments to them in order to generate predictions for the current election case. Additionally, none of these models were specifically designed with the current case study in mind.

#### 2.2.1 Election forecasting models

Several of the formal models we test (e.g., Quillien, 2020; Icard et al., 2017) simulate counterfactuals to an event as a proportion of their prior probability. In the current context, these counterfactuals are alternative ways in which the election could have unfolded. The prior probabilities of these counterfactuals vary: for instance, it is intuitively more likely for Biden to have lost Georgia than to have lost California. To quantify the prior probability of a given election outcome, we use election forecasts.^6^ Specifically, we used simulation data from two election forecasts developed by major news outlets: FiveThirtyEight (Silver, 2020) and The Economist (Heidemanns et al., 2020).

These forecasts combine various sources of information (e.g., demographic and economic variables, polling data) to predict the winner ahead of the election. For instance, the day before the election, FiveThirtyEight predicted an 89% chance that Biden would be elected president. These forecasts involve complex computations, which cannot be solved analytically, so they use Monte Carlo simulations. They simulate tens of thousands of possible election outcomes, in proportion of their estimated prior probability. For example, the FiveThirtyEight forecasting model estimated an 89% probability of a Biden victory because Biden won the presidency in 89% of these simulations. See Silver (2020) and Heidemanns et al. (2020) for more details about each forecasting model. The last versions of the forecasts can be accessed at https://projects.fivethirtyeight.com/2020‐election‐forecast/ and https://projects.economist.com/us‐2020‐forecast/president.

We downloaded the open‐access simulation data from the latest versions of the two election forecasts, last updated just before the election (accessible at https://projects.economist.com/us‐2020‐forecast/president, https://projects.fivethirtyeight.com/trump‐biden‐election‐map/simmed‐maps.json, shared under CC‐4 license, both datasets downloaded on November 9, 2020).

The simulation sets from FiveThirtyEight and The Economist contain 40,000 and 80,000 simulated election outcomes, respectively.^7^ For each simulated election outcome, the data contain the proportion of votes for a given candidate in each state. This allows us to compute, for that simulated election, the states where Biden won, and whether he won the presidency.

We now introduce the computational models of causal judgment we test. A fuller formal treatment is given in the Supplementary Information (https://osf.io/6jgez/). R code to implement the models is available at https://osf.io/r85tg/.

#### 2.2.2 Counterfactual effect size model

According to the CESM (Quillien, 2020), people are sensitive to two kinds of criteria when they make causal judgments. First, they care about what happened in the actual world. For example, one will not judge that event C caused an effect E if C did not actually happen, or if there was no way for C to have a causal influence on E in the current situation. Second, people are sensitive to the relationship between C and E across counterfactual situations.

The CESM does not attempt to directly model the influence of the first set of criteria (i.e., the criteria related to what happened in the actual world; see e.g., Halpern, 2016 for models that focus on these). Instead, it focuses on how the mind assesses the causal strength of events that meet these criteria. It holds that the causal strength of a cause C for an effect E is a measure of the “effect size” of C for E, across counterfactuals to the event.

The intuition is that causal strength is something like the correlation between C and E across counterfactuals. A correlation coefficient quantifies the statistical association between C and E regardless of whether C has a causal influence on E (e.g., thunder is correlated with lightning but does not cause it); the CESM defines a measure that is conceptually similar to a correlation coefficient, but is designed in such a way that it gives a score of 0 if interventions on C cannot have an effect on the value of E.

A correlation coefficient between C and E can be decomposed into two elements: a regression coefficient (the slope parameter in a linear regression predicting E from C), and the ratio of the standard deviations of C and E. The CESM follows this approach, but it computes a regression coefficient that can be given a causal interpretation (e.g., a regression coefficient that would be 0 for the effect of thunder on lightning).^8^

This regression coefficient quantifies the “average causal effect” of C on E across counterfactuals. By “causal effect” we mean, roughly, by how much the value of E would change in a given situation if an exogenous intervention changed the value of C by one unit. The average causal effect is additionally standardized by the ratio of the standard deviations of C and E. The standardization ensures that the measure behaves as an effect size (so that one gets the same causal effect regardless of the unit of measurement).

In what follows, we introduce the model in the context of the current election case. Note that we use the exact same model as described in Quillien (2020). The only aspect of our implementation of the model that is specific to the current case is the use of election forecasts to quantify the prior probability of counterfactuals.

To compute the actual causal strength of a state *S* won by Biden, the model proceeds as follows:

a) Across all simulations, compute the standard deviation (*σ_S_*) of the binary variable denoting whether Biden wins state *S*, the standard deviation (*σ_P_*) of the binary variable *P* denoting whether Biden wins the presidency, and the proportion of simulations where Biden wins state *S* (*Pr*(*S*)).
b) For each simulation, create a “twin simulation” by making an intervention setting *S* to a new, randomly sampled value (while holding all other states constant). That is, make Biden win *S* in the twin simulation with probability *Pr*(*S*) and lose with probability *Pr*(∼*S*). If the value of *S* is different between the simulation and its twin, compute the ratio of the change in the value of *P* between the two worlds to the change in the value of *S* (denoted *Δ_P_*/*Δ_S_*). For instance, if in the original simulation Biden wins state *S* and wins the presidency, and in the twin simulation he loses *S* and loses the presidency, then *Δ_P_*/*Δ_S_* = –1/–1 = 1.
c) Across all such pairs (i.e., a simulation and its twin simulation), compute the average value of *Δ_P_*/*Δ_S_*, then multiply this value by *σ_S_*_/_ *σ_P_*. This is the causal strength of *S* for *P*.

We computed causal judgments for two versions of the CESM: the first version uses the simulations from the forecast by The Economist, and the second version uses the simulations from the forecast by FiveThirtyEight.

#### 2.2.3 Other computational models

We found that many existing formal models of causal judgment could not make clear quantitative predictions about the current election case (see Supplementary Information https://osf.io/6jgez/). For example, some are only designed to account for intuitions about physical events (e.g., Wolff, 2007; Gerstenberg et al., 2021), whereas others are only designed for scenarios with a simpler causal structure (e.g., Morris et al., 2018). We were able to derive clear predictions for two other models of causal judgment.

The first model (Chockler & Halpern, 2004), which we will call the “Pivotality model”, quantifies the causal strength of an event as inversely proportional to its “distance from pivotality”. An event is pivotal for an outcome if the outcome would not have happened in the absence of that event. Consider, for instance, a committee voting whether to adopt a resolution, which needs a majority to pass. If 6 out of 11 committee members voted in favor, and the resolution was adopted, then each of those committee members was pivotal for that outcome, since changing the vote of any of them would mean the rejection of the resolution. By contrast, if seven committee members voted in favor, then each of them is one step away from having been pivotal to the outcome. Therefore, the model considers a member who voted “Yes” as very causal in the first scenario, but somewhat less causal in the second scenario (see Supplementary Information for details, and for derivation in the election case https://osf.io/6jgez/). Note that this model does not consider the prior probability of counterfactuals, so it does not use the simulation data from the election forecasts.

The second model is the “Necessity‐Sufficiency” model (Icard et al., 2017). The causal strength of an event is there defined as a function of the event's *necessity* for the outcome (whether the outcome would still have happened in the absence of the event), as well as the event's *sufficiency* strength (the extent to which the event is in general sufficient to bring about the outcome, across possible worlds). In the context of the election case, the actual causal strength of a state is mostly determined by its sufficiency strength (because according to the above notion of necessity, only California was necessary for winning the election). To compute the sufficiency strength of a state, we look at simulations where Biden lost that state and lost the presidency, and compute the proportion of such simulations where making Biden win that state would have made him win the presidency (see Supplementary Information for details https://osf.io/6jgez/). Also, according to the model, the extent to which a state's sufficiency strength influences causal judgment is modulated by the frequency with which people simulate counterfactuals where Biden wins the state. Here, we assume that this quantity is equal to *Pr*(*S*), the probability that Biden wins the state. We computed *Pr*(*S*) as the proportion of election forecast simulations in which Biden wins the state. As for the CESM, we computed causal judgments for two versions of the Necessity‐Sufficiency model, one for each election forecasting model. We also computed judgments for a “naïve” version of the model (which we will not consider further because it was a very poor match to the human data; see Supplementary Information).

Both the “Pivotality” and “Necessity‐Sufficiency” models have had some success in predicting human causal intuitions in other contexts (Lagnado et al., 2013; Gerstenberg et al., 2015; Langenhoff et al., 2021; Icard et al., 2017; Morris et al., 2019). Importantly (and similarly to the CESM), they are general enough in scope that we did not need to make ad‐hoc adjustments to them to derive quantitative predictions from them.^9^

We preregistered the exact quantitative predictions for each model (see https://osf.io/m3hgf). We did not use any free parameters when comparing any of the models to the human data.

The computational models we focus on are designed to model judgments about the cause(s) of singular events. However, other measures of causal strength that apply to different problems exist. For example, cognitive psychologists have developed computational models of the process via which people infer the strength of general causal relationships (e.g., Cheng, 1997); and statisticians have developed measures of causal strength to help researchers interpret the results of their empirical studies (see Holland, 1986; Pearl, 1999). These models were not designed for the problem we are interested in here–namely, predicting human causal judgment about singular events. However, it remains possible that when people make causal judgments about singular events, they co‐opt algorithms that are useful for other problems (such as causal inference). To explore this possibility, we additionally test three prominent models of causal strength: Power‐PC (Cheng, 1997), the Probability of Necessity of Sufficiency (Pearl, 1999), and two versions of Delta‐P (Jenkins & Ward, 1965). We describe these models in the Supplementary Information (https://osf.io/6jgez/).

## 3 Results

**Fig 1:**
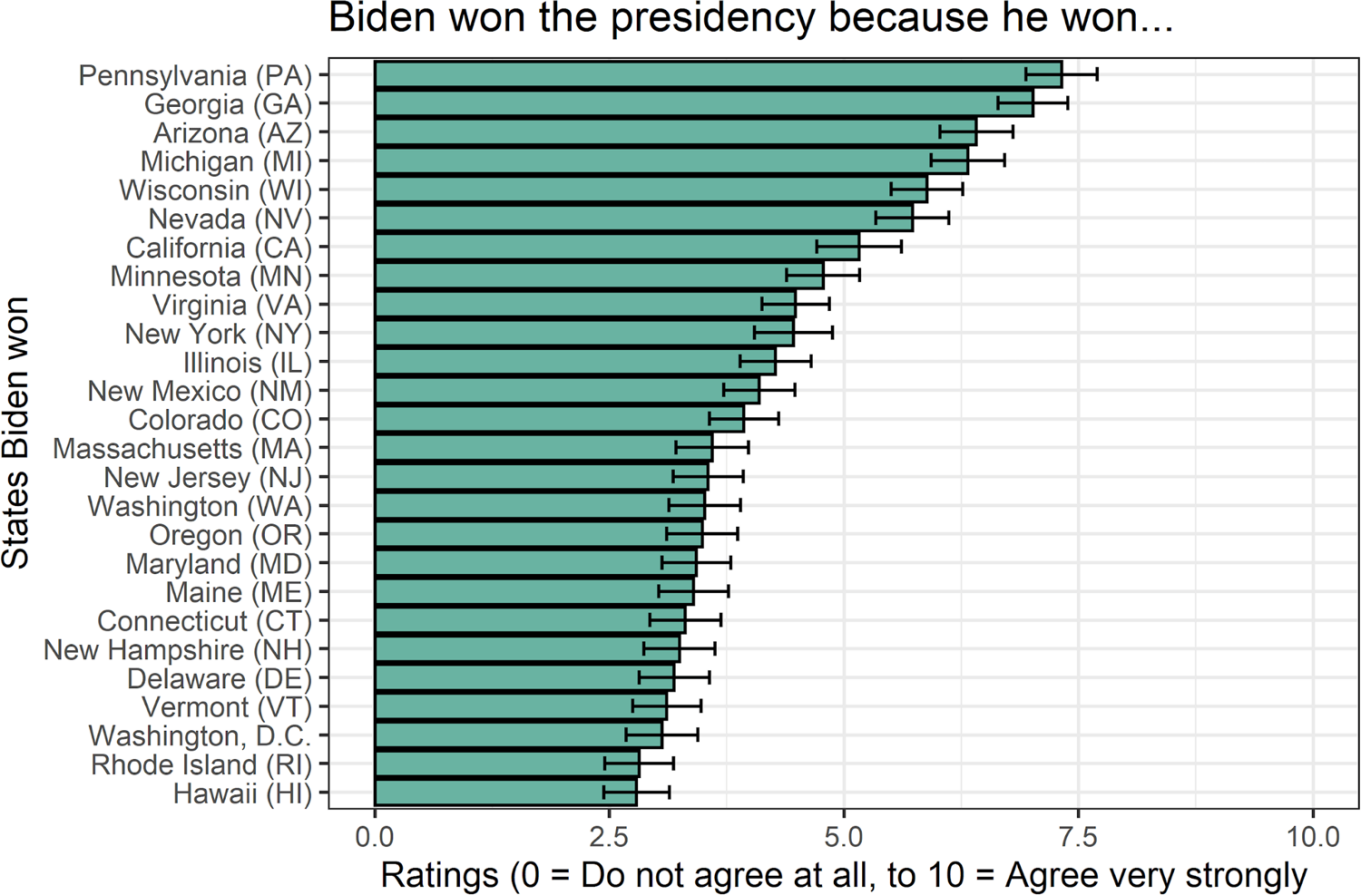
Average human causal ratings, for each of the 26 U.S. states Biden won. Error bars are 95% CIs.

The average causal ratings made by participants for each state are shown in Fig. 1. Fig. S2 shows the distribution of individual causal judgments for each state. Data are available on the Open Science Framework at https://osf.io/r85tg/.

### 3.1 Does the CESM predict human causal judgments?

**Fig 2:**
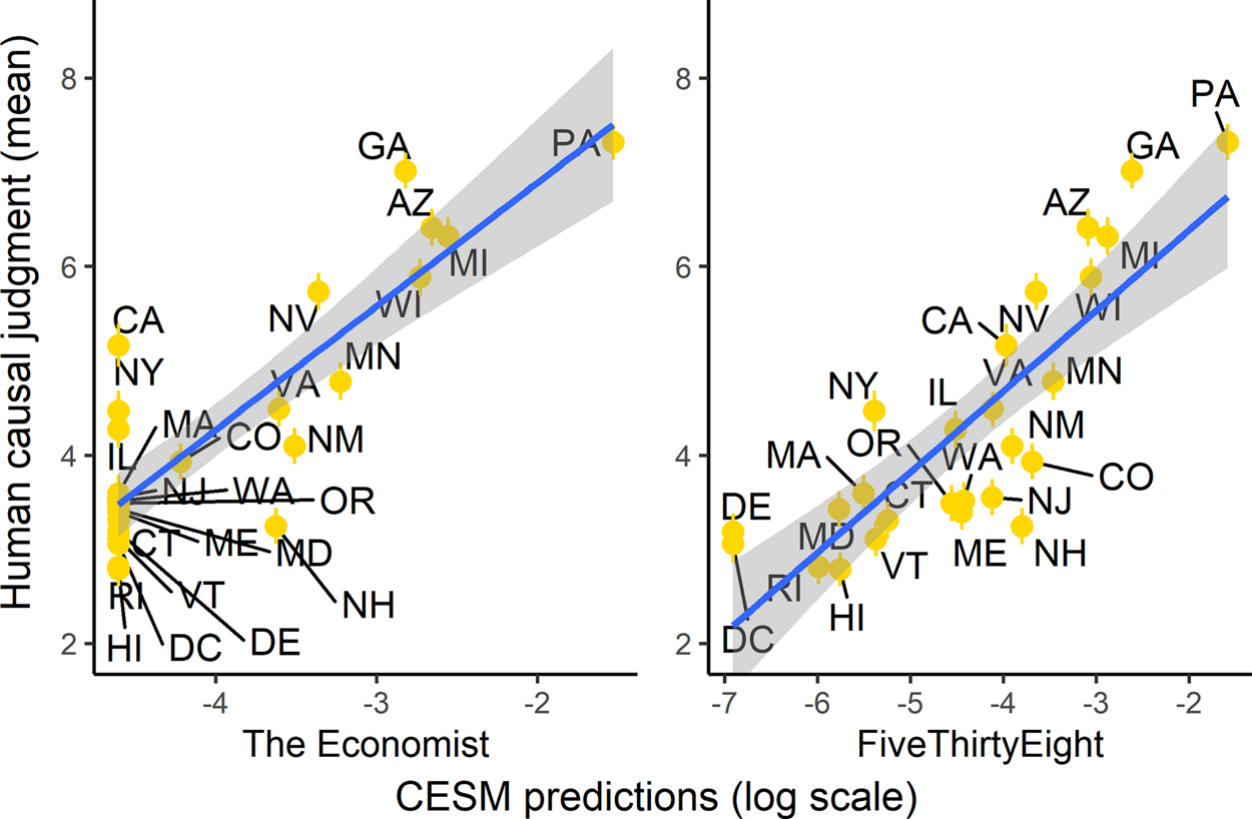
Correlation between the CESM and mean human causal judgments, across states. Left and right plots show the versions of the CESM calibrated with simulations from The Economist and FiveThirtyEight, respectively. Error bars represent standard error of the mean. Note that the fact that values on the x‐axis are negative is simply an artifact of the use of a log scale.

**Fig 3:**
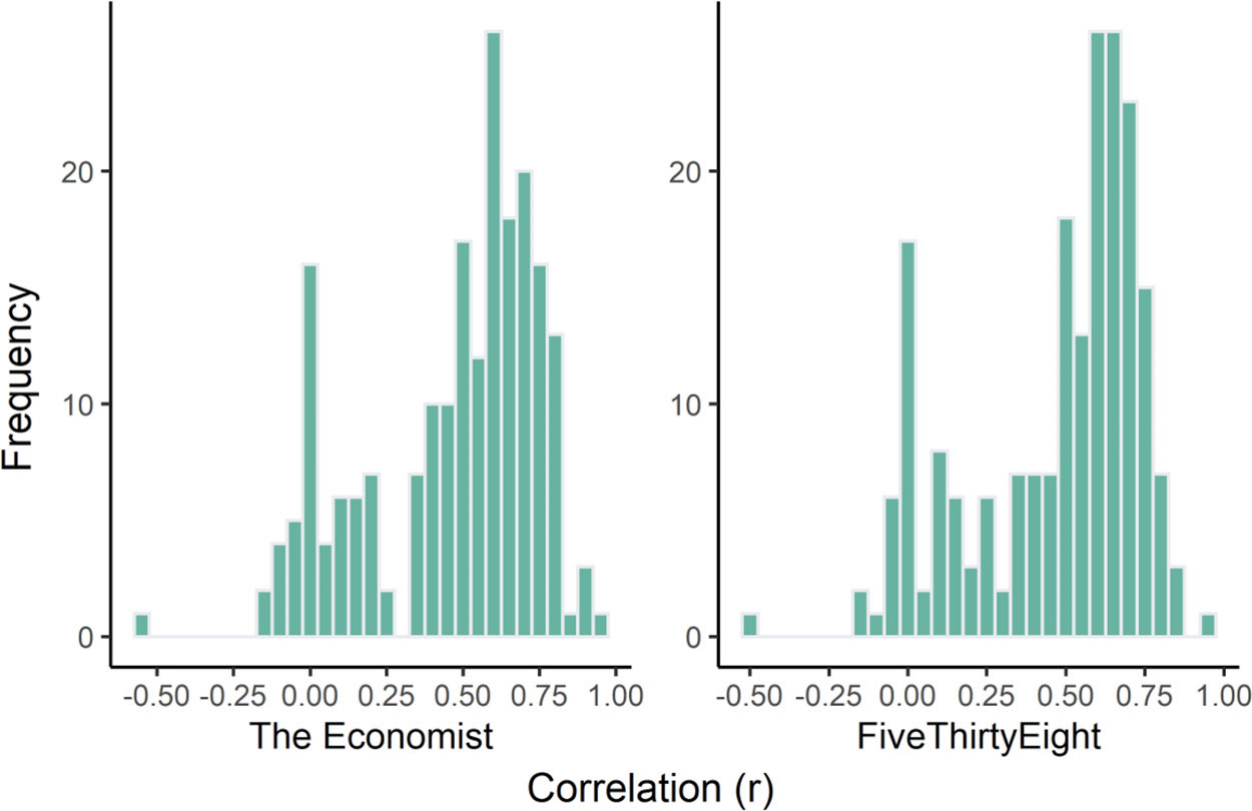
Individual‐level fit between judgments made by the CESM and human causal judgments. For each participant, we computed the correlation between the causal ratings made by the participant and the causal ratings made by the CESM. The histograms show the distribution of these correlation scores for the version of the model calibrated with The Economist (left) and FiveThirtyEight (right).

Yes. Across states, causal judgments made by the CESM were highly correlated with mean human judgments. This was true whether we compared human judgment to the version of the model calibrated with simulations from The Economist or FiveThirtyEight: *r*(24) = .77 for both versions of the model, *p*s <.001; see Fig. 2.^10^ A similar close fit is revealed when examining the individual‐level correlations. Here, for each participant, we computed the correlation between the causal ratings made by the participant and the causal ratings made by the CESM. The median correlation between a participant's ratings and the computational model ratings was *r*(24) = .55 for both versions of the model. See Fig. 3.

To get some intuitive understanding of the judgments the CESM makes, consider California and Georgia. California has many electoral votes, so in the simulated elections where Biden loses California, he often loses the presidency as a result. But these scenarios are rare, because California is a Democrat stronghold; therefore, across simulations in FiveThirtyEight, whether Biden wins or loses California is only moderately correlated with the election outcome; in The Economist, which gives a Republican victory in California an even lower chance, this correlation is even weaker.

The model gives high scores to states which combine a relatively high number of electoral votes with a relatively high outcome uncertainty. For example, Georgia has 16 electoral votes (tied for eighth highest) and FiveThirtyEight gave Biden a 58% chance of winning in Georgia. As such, across simulations, there is a lot of variation in the outcome in Georgia, and in many simulations, the outcome of the presidential election depends on the outcome in Georgia. In other words, the outcome in Georgia is highly correlated with the outcome of the presidential election.

### 3.2 Do other models of causal judgment better account for the human data?

**Fig 4:**
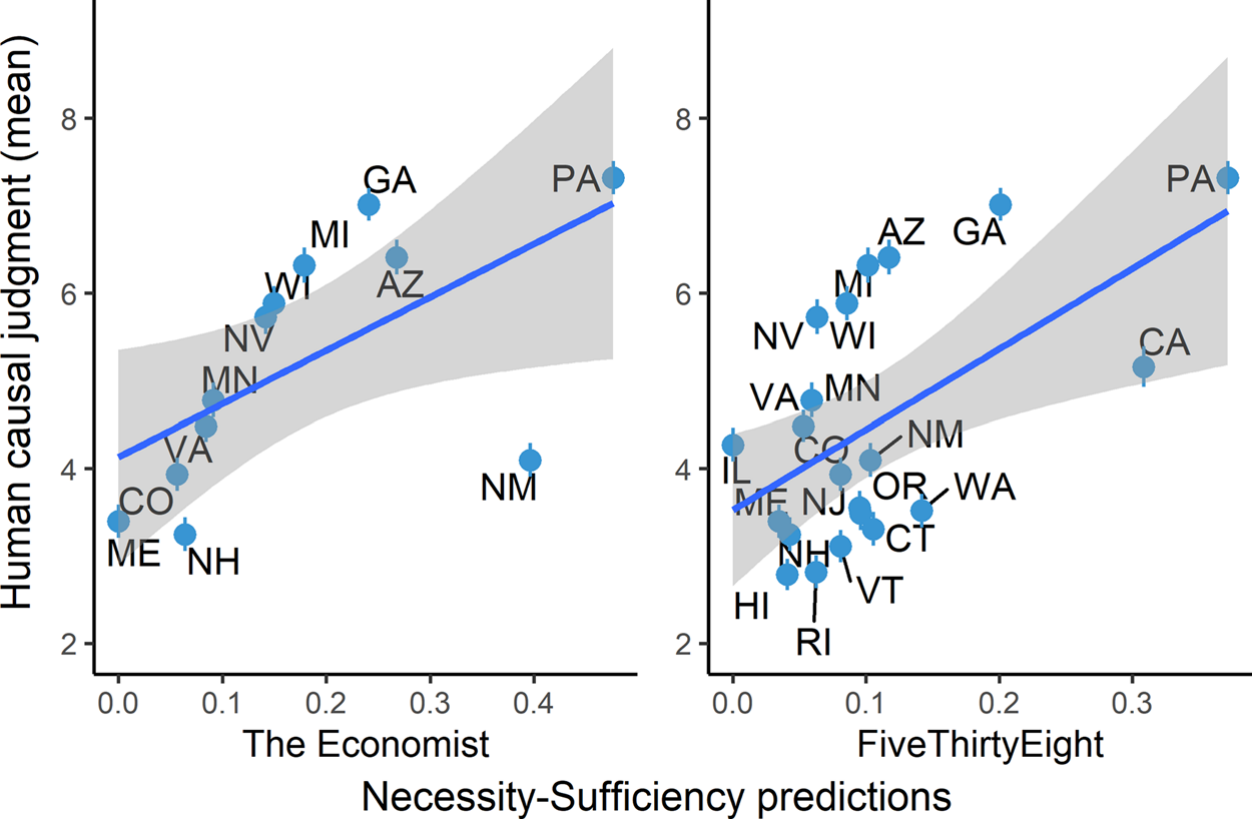
Correlation between the Necessity‐Sufficiency model and average human causal judgment, across states. Error bars represent standard error of the mean. Left and right plots show the versions of the model calibrated with simulations from The Economist and FiveThirtyEight, respectively.

No. Causal judgments made by the Necessity‐Sufficiency model correlated with human judgments at *r*(10) = .62, *p* = .03 for the version calibrated with The Economist and *r*(19) = .57, *p* = .007 for the version calibrated with FiveThirtyEight. See Fig. 4.

We note that computing causal ratings for the Necessity‐Sufficiency model was impossible for some states as this requires looking at simulations where Biden lost that state and lost the presidency (and we need to look at such simulations in order to compute the “sufficiency strength” of the state). When generating preregistered causal ratings for the Necessity‐Sufficiency model, we excluded any state for which we had fewer than 10 simulations where Biden lost that state and lost the presidency (e.g., there were very few simulations where Biden lost New York and lost the presidency). As a result, we could only compute ratings for 12 states (out of 26) for the version of the model calibrated with The Economist and 21 states for the version calibrated with FiveThirtyEight. Therefore, for adequate comparison, we also computed the correlation between the CESM and human judgments on these subsets of states. CESM ratings were correlated with human judgments at *r*(10) = .74, *p* = .006, for the version of the model calibrated with The Economist and *r*(19) = .75, *p* < .001 for this version calibrated with FiveThirtyEight. The CESM still outperformed the Necessity‐Sufficiency model when looking at this subset of states.

**Fig 5:**
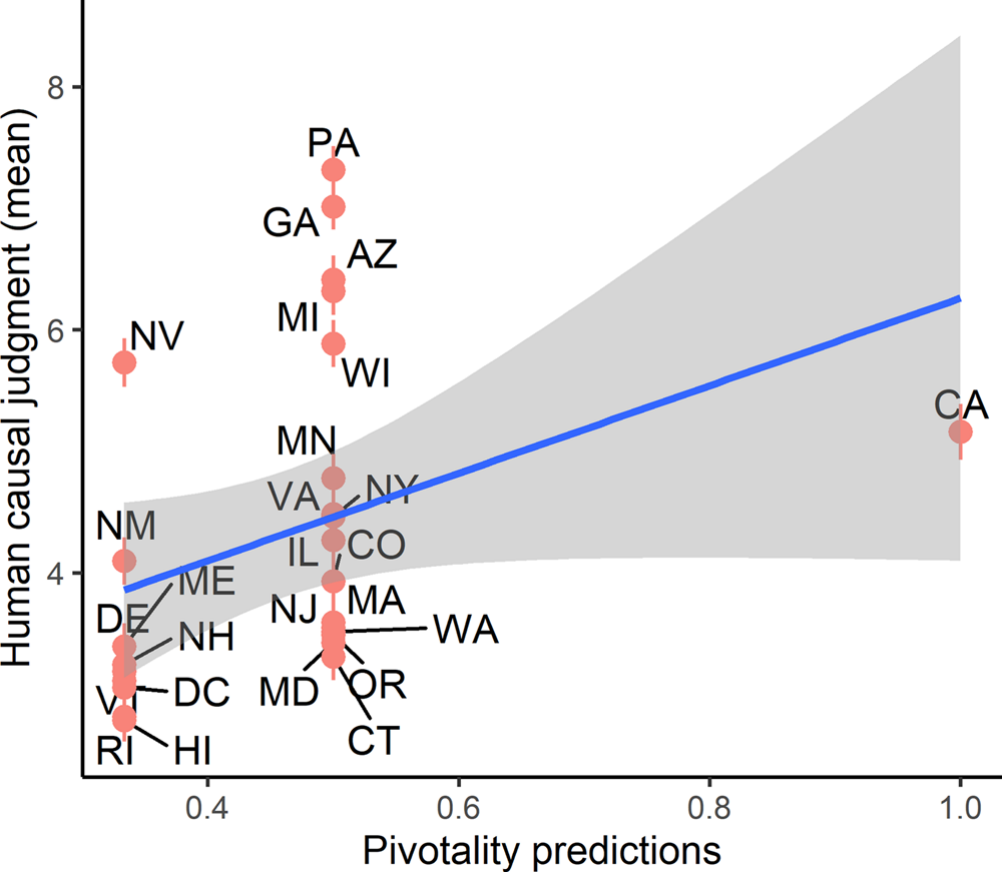
Correlation between the Pivotality model and average human causal judgment, across states. Error bars represent standard error of the mean.

Causal judgments made by the Distance‐From‐Pivotality model correlated with human judgments at *r*(24) = .36, *p* = .07. See Fig. 5.

**Fig 6:**
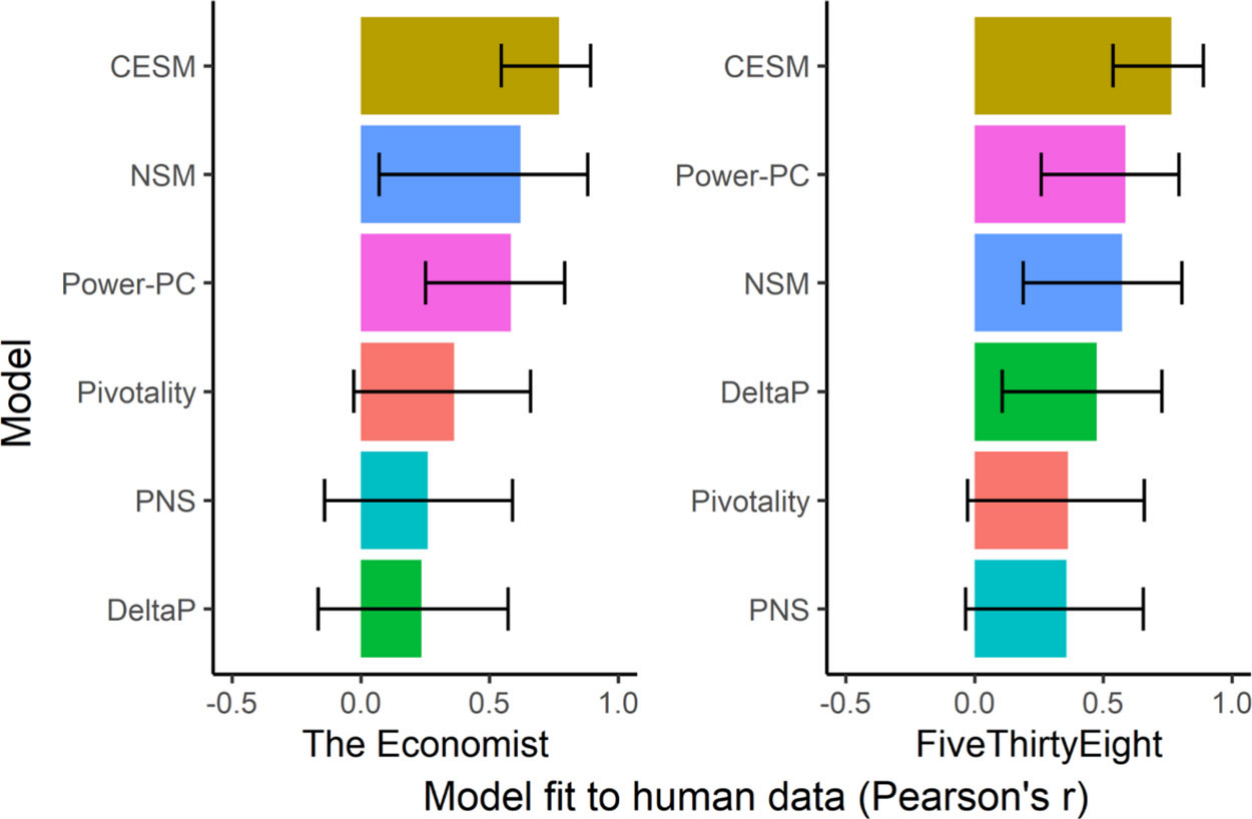
Fit of the CESM and each of the other models tested here to the human data, as measured by the correlation (Pearson's *r*) between model predictions and mean human causal judgment, across states. NSM, Necessity‐Sufficiency Model; PNS, Probability of Necessity and Sufficiency (note that this is equivalent to the version of Delta‐P that conditions on interventions; see Supplementary Information for details). Note that the correlation for the Pivotality model is identical between the left and right panels because this model does not use data from election forecasts. Error bars represent 95% confidence intervals.

None of the additional (exploratory) models we tested (Delta‐P, Power‐PC, and PNS) fit the human data as well as the CESM. Paired permutation tests (see Supplementary Information) show that either version of the CESM (i.e., the versions calibrated with FiveThirtyEight and The Economist) had a better fit to the human data than all other models (preregistered and exploratory) that we tested (*p*s < .001). See Fig. 6 for correlations (Pearson's *r*) between model predictions and mean human causal judgment, across states, for the CESM and each of the other models tested here.

### 3.3 Are these results a simple effect of prior probabilities (unexpected events)?

No. The prior probability of an event is known to influence causal judgments (Hart & Honore, 1985; Kahneman & Miller, 1986). Could it be that the CESM has a good fit to human judgments simply because it tends to assign higher causal strength to states that Biden won but was less likely to win? CESM judgments were moderately negatively correlated with the prior probability of Biden winning a state (*r*(24) = –.52, *p* = .006 for FiveThirtyEight; *r*(24) = –.33, *p* = .10 for The Economist). Yet, when controlling for prior probability in a multiple linear regression, both versions of the CESM were still significantly correlated with human judgments, β = 0.77, *p* < .001 (The Economist) and β = 0.56, *p* < .001 (FiveThirtyEight). Thus, the CESM does more than simply formalizing the fact that people give high causal scores to unexpected events.

### 3.4 Are these results a simple effect of the number of electoral votes?

No. Intuitively, states that have more electoral votes make a larger contribution to a candidate's victory. Does the CESM predict human judgments simply because it formalizes this intuition? CESM predictions were only weakly (and nonsignificantly) correlated with the number of electoral votes in a state (*r*(24) = .21, *p* = . 31 for FiveThirtyEight and *r*(24) = .14, *p* = .50 for The Economist). Controlling for the number of electoral votes in a state in a multiple linear regression, both versions of the CESM were significantly correlated with human judgments, β = 0.73, *p* < .001 (The Economist) and β = 0.71, *p* < .001 (FiveThirtyEight). Therefore, the predictive power of the CESM is not simply due to the fact that it gives higher causal scores to states with more electoral votes.

We also computed multiple regressions where we control for both prior probability and number of electoral votes at the same time. CESM predictions remained significantly associated with human ratings, β = 0.72, *p* < .001 (The Economist) and β = 0.71, *p* < .001 (FiveThirtyEight).

### 3.5 Do these results hold across political party affiliation?

Yes. For each state, we computed the average causal rating for participants who indicated having voted for Biden (*N* = 158) and for Trump (*N* = 29). Across states, the correlation between causal judgments from Biden voters and Trump voters was nearly perfect: *r*(24) = .97, *p* < .001.

Trump voters did give overall significantly lower causal ratings (*M* = 2.88) than Biden voters (*M* = 4.59), *t*(49.3) = 4.73, *p* < .001. Data collection took place about a month after the presidential election. At that time, then‐President Trump was spreading misinformation about the election outcome. The mean difference in causal ratings between Trump and Biden voters is likely due to the fact that most Trump voters in our sample did not believe that Biden won the election legitimately (21 out of the 29 Trump voters reported believing this). Nevertheless, their relative ranking of the causal strength of the states was strikingly similar to that of Biden voters. We find the same invariance when we group participants by partisan identification (*r*(24) = .95, *p* < .001) or belief in the election's legitimacy (*r*(24) = .96, *p* < .001).

### 3.6 What explains differences between the models in predicting human causal judgments?

In exploratory analyses, we find that one possible reason the CESM fits the human data in this case study better than the other two main models we tested (Necessity‐Sufficiency and Pivotality) is that when making causal judgments, like humans and unlike those other two models, it assigns higher weight to outcome probability than to number of electoral votes.

More formally, we ran a multiple regression predicting people's causal judgments as a function of the prior probability of a Biden victory in each state and the number of electoral votes in each state, using the simulations from FiveThirtyEight. We ran a similar analysis using the simulations from The Economist. Then, we repeated this analysis for the CESM, Necessity‐Sufficiency, and Pivotality models. Results showed that for human participants and the CESM, prior probability predicted judgments about the causal importance of each state more strongly than the number of electoral votes; the Necessity‐Sufficiency model and the Pivotality model showed the opposite pattern (Table 1).

**Table 1:** Standardized beta coefficients from multiple regression analyses predicting causal judgment in a state, averaged across states, as a function of the prior probability of a Biden victory in that state, and the number of electoral votes in that state

	β_prob_ FiveThirtyEight	Β_votes_ FiveThirtyEight	β_prob_ The Economist	Β_votes_ The Economist
Humans	–0.69***	0.41**	–0.59***	0.38*
CESM	–0.52**	0.20	–0.32	0.12
Necessity‐Sufficiency model	–0.30	0.65***	–0.11	0.47
Pivotality model	0.01	0.90***	–0.01	0.90***

^*^ *p* < .05.^**^ *p* < .01.***p < .001.

These results can be understood intuitively, for example by comparing California with states such as Arizona and Georgia. Both human participants and the CESM hold that AZ and GA are more causally important than CA–because the election outcome in AZ and GA is more uncertain than in CA–despite the fact that CA is the state with the most electoral votes. By contrast, the Necessity‐Sufficiency model and the Pivotality model rank CA above GA and AZ.

These results can also be understood by looking at the underlying logic of each model. The CESM gives low causal scores to states where Biden was guaranteed to win, because the outcome in such states is almost always the same across simulations; this low outcome variability means that the outcome in those states does not correlate highly with the general election outcome. For example, almost all simulations where Biden wins the election are simulations where he wins in California, but the same can be said for almost all simulations where Biden loses the election.

According to the Necessity‐Sufficiency model, causal judgment about a state mostly depends on whether winning the state is sufficient, in general, for winning the election. Formally, a state is sufficient for victory to the extent that, in simulations where Biden loses the state and loses the election, he would have won the election if only he had won the state (see Supplementary Information for more details). Sufficiency strength depends mostly on the state's number of electoral votes, and therefore, the model tends to weigh electoral votes more highly than participants do.

Finally, by design, the Pivotality model depends entirely on electoral votes, because it does not take probabilistic information as input.

## 4 General discussion

Of the many factors that lead to an event, how do people decide which were the most causally important? Researchers have suggested that people make judgments about the causes of an event by considering “counterfactual worlds” (i.e., alternative ways in which the event could have happened). Computational models based on this proposal have been very successful in predicting human causal judgments in simple experiments, but have never been tested in complex real‐world cases.

Here, we provide such a test using the case study of the 2020 U.S. presidential election. We asked participants about the causal contribution of different states to Biden's victory, and compared their judgments to those made by several computational models.

We find that counterfactual models of causal judgment provide a good fit to human intuitions. The CESM (Quillien, 2020), in particular, closely tracked human judgments, and did so significantly better than other models we tested. The Necessity‐Sufficiency model (Icard et al., 2017), another counterfactual model, also predicted human intuitions well.

The CESM closely tracked human causal judgments even when controlling for the prior probability of Biden winning each state and the number of electoral votes in each state; and it was completely invariant to factors such as how participants voted and their partisan identification.

Our findings are deeper than merely that participants knew which states were “swing states.” Participants, indeed, appeared to know which states were most “decisive” for the election outcome (i.e., most correlated with the outcome across counterfactuals), but most importantly, they also considered such “decisiveness” as crucial for causal judgment—just as predicted by the CESM. Consider that many other criteria, such as the number of electoral votes in a given state, could in principle have regulated causal attributions instead. Yet, participants judged Wisconsin, with less than a fifth of California's votes, as more causally important.

### 4.1 Scope of the current research

The current work is concerned with the computational level of analysis of the mind (Marr, 1982). When the human mind assigns a cause to an event, which information‐processing problem is it solving? We tested the idea that mechanisms for causal judgment are designed to, at least in part, compute an “effect size” measure of the causal dependence of an effect on a cause across counterfactuals (Quillien, 2020). By contrast, our data cannot tell us what specific algorithms and representations participants used in their causal judgments.

The computational models implemented here are idealized benchmarks. People probably represent the possible outcomes of a U.S. election in a much less sophisticated manner than professional election forecasting models. It is also unlikely that people simulated tens of thousands of possible alternative election outcomes, as FiveThirtyEight and The Economist did. At the algorithmic‐representational level of Marr's hierarchy (1982), a full account of causal judgment in the current task would have to answer the following two questions.

First, how did people construct their causal model of the U.S. 2020 presidential election? People could have done so in several (nonmutually exclusive) ways. For example, people have a general sense of which states lean Democrat versus Republican, and could have used this to predict how those states are likely to vote. People may have estimated the number of electoral votes in a given state by extrapolating from the state's population, or even by assuming that states they are most familiar with have more residents and, therefore, more votes (Gigerenzer & Goldstein, 2011). People may have generalized from what they remembered about past elections, and they may have followed what journalists covering the campaign said about the likely outcomes in different states. Since these data were collected after the election was called, it is also possible that participants used information acquired during or after the election to revise their causal model of the election. For example, for states where the Democratic and Republican candidates had similar numbers of votes, participants might have inferred that the outcomes in those states were a priori uncertain.

Second, how did people use their causal model of the election to compute the index of causal strength specified by the CESM? We note that our computational‐level proposal is not in principle committed to the idea that people actually need to perform any simulations of the election outcome. For instance, it may be possible for the mind to use algorithms that compute a measure of effect size over possible counterfactuals even without actually generating these counterfactuals. In fact, researchers who develop counterfactual accounts of causal judgment often do exactly this. When they generate the predictions of a computational model in a simple setting, they typically do not sample counterfactuals from that setting but derive an analytic expression that expresses the causal measure that a simulation‐based algorithm would converge on (Icard et al., 2017; Morris et al., 2018; Quillien, 2020). In practice, it is likely that people generate a small number of coarse‐grained simulations in order to compute this index in a resource‐rational way (see Vul et al., 2014).

We used forecasting models as a proxy for people's mental representation of possible outcomes of the election. Maybe these forecasts also had a causal influence on people's representations, because participants followed the forecasts or heard about them from friends or news media. We note, to preempt misunderstandings, that this poses no difficulty to our account. Remember that we are not interested in how people constructed their causal representation of the election, but in how they derive a causal judgment from this representation. Forecasting models do not on their own determine a measure of causal judgment. We used the simulated election outcomes from these forecasts as merely an *input* to a *computation* that generates a measure of causal strength.^11^

There are many different possible models of this computation, and the problem is to find which model gives a close approximation of people's causal judgments. For instance, people may have computed the causal strength of a state as the probability that Biden wins the election, given that he won the state, minus the probability that Biden wins the election, given that he did not win the state. This measure is popular, both as a psychological model of causal reasoning (where it is called Delta‐P; Jenkins & Ward, 1965) and as a measure of causal strength in a wide variety of fields, such as political science, epidemiology, and econometrics (Gelman et al., 2002; Schafer & Kang, 2008; Holland, 1986; Sprenger, 2018). But it was not able to accurately reproduce people's causal judgments in the current study. In fact, we tested many such prominent measures of causal strength, and the CESM had a significantly better fit to the human data than all of them (Fig. 6).

### 4.2 Limitations, implications, and future research

The current study is correlational and we cannot, therefore, definitively rule‐out all alternative interpretations of our findings. For example, maybe the CESM provides a good account of the data because it tells us about the way that experts and journalists commented on the election campaign, but does not directly tell us about the way laypeople make causal judgments. Under this hypothesis, participants may have passively repeated what they heard experts say about the importance of a given state, and the CESM only provides a description of how experts quantified a state's importance when making predictions about the election's outcome. We note that even this extreme hypothesis would require a counterfactual approach to causal judgment. An explanation of the type “people heard that state X was going to be important, and this makes them select that state as the cause” is not on its own a computationally adequate explanation. To be complete, the explanation needs to specify: (1) how people interpret a prediction about the “importance” of an event, (2) why they should see it as relevant to a causal judgment after the fact, and (3) why they should see it as more relevant than other information such as, for example, the number of electoral votes in a given state. Counterfactual models like the CESM provide these missing pieces, because they hold that people make causal judgments by using some of the cognitive mechanisms they use to make and interpret predictions.

More importantly, our correlational results are surprisingly convergent with the results from a wide range of tightly controlled experimental studies, where people could not rely on any external opinions about the “importance” of a given factor (Icard et al., 2017; Gerstenberg & Icard, 2020; Kominsky & Phillips, 2019; Henne et al., 2019; Quillien & German, 2021; Morris et al., 2019).

For example, in Morris et al. (2019; see O'Neill et al., 2021 for an extended replication), participants had to make causal judgments about the outcome of a simple casino game where a player randomly draws balls of different colors from two urns. The proportion of balls of the winning color in each urn had a large influence on participants’ judgments of why the player won the game. Furthermore, the direction of this effect changed depending on the rules of the casino game. Under one set of rules, the more balls of the winning color there were in an urn, the more the participants said that the player won because he drew a ball of the winning color from that urn. Under another set of rules, the effect ran in the opposite direction. Participants’ answers exhibited many other subtle patterns, which were not explained under existing theories of causal judgment (Morris et al., 2019). Subsequent analysis showed that all these effects could be parsimoniously explained by the hypothesis that participants computed the correlation, across counterfactuals, between drawing a ball of a given color and winning the game (Quillien, 2020). Neither the experimental studies, nor the current correlational findings, are definitive on their own, but they converge on a strikingly similar picture of human causal judgment.

We hope that the present work encourages other researchers to conduct further studies of causal judgment “in the wild.” The use of real‐world case studies is methodologically challenging, because researchers need some way to model people's internal representation of the relevant causal structure. Here, we were able to do so by using election forecasts, but other resources might exist that would provide similar opportunities for other case studies.

Data from such studies would be useful, among other things, for informing comparison between different counterfactual models of causal judgment. We find that the CESM fit the human data in the present case study better than other models explored, and that this might be in part because the CESM is more sensitive to outcome uncertainty than these other models. But this difference in model fit could also be, at least in part, explained by idiosyncrasies of the current case study. For example, the election forecasts we used were designed prior to the election, but participants could have also informed their causal judgments by new information they learned on election day and after. For example, states like Georgia, Arizona, and Nevada were the focus of intense attention in the days following the election, which might have increased the weight participants placed on these states in making their causal judgments; it is in principle possible that this focus uniquely advantaged the CESM compared to the other models investigated here. Thus, it would be particularly interesting to study causal judgment in a real‐world situation where it is possible to model the information that participants gain prior to, during, and after the actual event. Also, the current election case study has the particularity that many commentators tried to predict the outcome before the event, and it might be interesting to conduct real‐world case studies where most of the commentary instead happens after the event.

Future research could test more complex versions of the CESM. For instance, the simulation data we used contain only counterfactual worlds which were simulated without looking at the actual outcome of the election. In general, people tend to sample counterfactuals as a function of their similarity to what actually happened, in addition to their prior probability (Lucas & Kemp, 2015)—this could be incorporated in more detailed versions of the CESM.^12^

Finally, the present work illustrates one way of understanding why causal judgment involves counterfactual thinking. We were able to predict human causal intuitions by simply letting the CESM look at election forecasts and the states Biden won. However, election forecasts are designed for prediction, not causal judgment; that they could be used to model causal judgment suggests a deep connection between the two cognitive processes.

Specifically, causal judgment about an event may, at least in part, be designed to encode the most important information contained in the causal model we would have used to predict the event. For instance, by telling someone “Biden won the election because he won in Pennsylvania,” speakers allow listeners to infer that Pennsylvania might be decisive for the next election as well.

Gerstenberg and colleagues (2021) have reported similar results in the domain of intuitive physics. They were able to model the causal judgments that humans make about simple physical events by assuming that participants use a predictive physics engine to simulate counterfactuals. Here, we show that causal judgment in a social domain, with a much richer set of variables, can be modeled in the same spirit.

## 5 Conclusion

When explaining what caused a complex real‐world event such as a forest fire, people select certain factors (e.g., a lightning strike) while ignoring others (e.g., oxygen in the air). We suggested that such intuitions about causes of events are regulated by estimates of the causal strength of the different factors, calculated by implicitly considering alternative ways in which the event could have happened. As a whole, the present findings—using the 2020 U.S. presidential election as a case study—support this counterfactual approach to human causal judgment.

## Funding

The authors have no funding to report.

## Conflicts of interests

The author(s) declared have no conflicts to disclose.

## Supporting information

Fig. S1: Correlation between the simplified CESM predictions and average human causal rating, across states.
Fig. S2: Frequency of causal ratings, broken down by state.

Supplementary Material

## Notes

1 The proposal that people use probabilistic causal models is often coupled with the assumption that they generate accurate predictions from these models–a corollary that is not always consistent with evidence (Ludwyn‐Peery et al., 2021). But the general point we are making here does not depend on this assumption.

2 As another example, when we try to predict and explain the behavior of other people, we rely on a rich causal model of the minds of intentional agents, which holds representations such that perception causes beliefs, beliefs and desires jointly cause intentions, and intentions cause actions (Leslie, 1994; Lucas et al., 2014; Baker et al., 2017; Quillien & German, 2021).

3 Many factors influence the counterfactuals that people generate. In the current study, we focus on one such factor–prior probability–but we note that the CESM can account for the influence of a wide range of other factors on causal judgments. For example, people tend to generate counterfactuals where agents do not violate norms, are more likely to mentally mutate recent events compared to old ones, and are more likely to mutate actions rather than omissions (Byrne, 2016). If we plug these assumptions into the CESM, it predicts the exact way in which these factors (prescriptive norms, recency, and action‐vs.‐omission) influence causal judgment (as documented in Icard et al., 2017; Henne et al., 2019, 2021b).

4 In the scenario where Bob needs to pass both history and mathematics to graduate, it is easy to imagine Bob passing history, but failing mathematics, and not graduating. Therefore, whether Bob passes history is not very diagnostic as to whether he graduates. By contrast, the situations where Bob passes mathematics are almost always situations where he graduates (because we tend to assume that he passes history; there will be situations where he passes mathematics but fails history, but these are very implausible and, therefore, do not contribute much to causal judgment).

5 The CESM also predicts that (1) when people evaluate the causal strength of an event C, they will be influenced by the prior probability of other events that also contributed to the outcome, and (2) the direction of that effect depends on the type of causal structure. These predictions are consistent with the empirical evidence (Kominsky et al., 2015; Morris et al., 2019).

6 We also use election forecasts as an input to the measures of causal strength we test in our exploratory analyses: Delta‐P, Power‐PC, and PNS.

7 The Economist would usually run 20,000 simulations when updating its forecast in the weeks before the election, but used 80,000 simulations for its final forecast; G. E. Morris, personal communication.

8 Thus, the CESM computes an index of causal strength that is often quite close to a correlation coefficient. Indeed, in the current case, the CESM measure is approximately equivalent to the correlation between a Biden victory in state *S* and a Biden victory at the presidential election (*P*), across simulations. The fit between a simple correlation model and the CESM judgments are *r*(24) = .83 when using simulations from The Economist, and *r*(24) = .76 when using simulations from FiveThirtyEight. We say more about similarities and differences between a simple correlation model and the CESM in the Supplementary Information (https://osf.io/6jgez/).

9 One could also construct an infinity of ad‐hoc models that rely on specific features of this particular case study. Some of these models will inevitably be a near‐perfect match to the human data, because of overfitting. However, such a project would be an exercise in curve‐fitting rather than an investigation of the general logic of causal judgment: models that rely on idiosyncratic features of the current case would be unable to make predictions about human causal intuitions in other contexts.

10 When looking at the data, we saw that human ratings grow as the log of the model predictions. Therefore, to make visually comparing the CESM to human judgments easier, we plotted the data on a logarithmic scale. But since we have no strong theoretical rationale for expecting a log relationship, we conduct all statistical analyses with untransformed data.

11 This point is easy to overlook because of instinct blindness, the tendency of psychologists to fail to notice the necessary existence of computations that they do not have conscious access to (Cosmides & Tooby, 1994). Because people make causal judgments effortlessly, it is tempting to think that these judgments are transparently available from one's causal representation of the world. But in fact they require an additional computation.

12 In the current context, similarity to what actually happened and prior probability are very closely related. It might have been important to take the actual world into account if very unlikely events (e.g., Biden losing in New York) had occurred on election night.
