# Supplementary material for: Causal Judgment in the Wild: Evidence from the 2020 U.S. Presidential Election
Source: Cogn Sci. 2022 Feb 5;46(2):e13101. doi: 10.1111/cogs.13101 (PMC10015993; doi:10.1111/cogs.13101)
Supplement: Supplementary file 1 — Fig. S1: Correlation between the simplified CESM predictions and average human causal rating, across states. Fig. S2: Frequency of causal ratings, broken down by state. [file COGS-46-0-s002.pdf]

Supplementary Information for 'Causal judgment in the wild – evidence from the 2020 US presidential election'

Contents

1 Computational models **3**

1.1 General Framework 3

1.2 Pivotality model 5

1.3 Necessity-Sufficiency model 7

1.4 Counterfactual Effect Size Model 10

1.5 Paired permutation tests 12

2 Other models of causal cognition **13**

2.1 Models of causal induction 13

2.2 Process theories 14

2.3 Dependence theories 15

2.4 Exploratory analyses with other measures of causal strength . 17

2.4.1 Delta-P 17

2.4.2 Probability of Necessity of Sufficiency (PNS) 18

2.4.3 Power PC 19

3 'Simple' versions of the Necessity-Sufficiency model and CESM 20

4 Additional figures 23

4.1 Histograms of causal ratings, broken down by state 23

5 Modified U.S. political knowledge quiz 23

1 Computational models

1.1 General Framework

We first introduce the concrete concepts needed for reasoning formally about causality in the context of the current paper. Note that these concepts have broader and more abstract definitions too. See Pearl (2000), Halpern (2016), and the supplementary information in Quillien (2020) for discussions of these.

We model the **causal structure** of the US presidential election in the following way. There are 51 random variables (the 50 US states + the District of Columbia) that can take the value 1 (if Biden wins the state) or 0 (if Biden loses the state). There is also a random variable P for "Biden wins the Presidency" whose value is a deterministic function of the other variables: 1 if Biden wins enough states to get 270 or more electoral votes, and 0 otherwise.

We call a **world** a particular assignment of values to variables in the causal structure. Notably, the **actual world** is what actually happened: it is a setting of the causal structure where all the states that Biden did win in the 2020 election have value 1, the other states have value 0, and Biden wins the presidency. But we also consider **possible worlds** where the variables can take other values – for instance, a world where Trump lost in Florida. Importantly, all worlds (possible and actual) must be consistent with the rules of the causal structure (for example, there cannot be a world where Biden wins less than 270 electoral votes but wins the presidency).

Two election forecasting models are used here: FiveThirtyEight (Silver, 2020) and The Economist (Heidemmans, Gelman & Morris, 2020). Election forecasting models simulate possible worlds (i.e. possible outcomes of the election) in proportion to their likelihood. In other words, a forecasting model

implicitly defines a probability distribution over possible worlds. The two forecasting models used here operate over the same causal structure, but assign slightly different probability distributions over possible worlds. Both forecasting models also assume that states are not statistically independent from each other. For instance, in the FiveThirtyEight model, there is a correlation across possible worlds of r =.79 between the value of Wisconsin and that of Michigan. That is, in worlds where Biden wins in WI he is disproportionately likely to also win in MI.

As modelers, we can modify a (possible or actual) world by performing **interventions** on variables. An intervention is the act of setting a variable to a new value of one's choice. For instance, one can intervene on WI by setting WI=1 (i.e. making Biden win in Wisconsin), regardless of the current value of the variable. Interventions are not constrained by the probability distribution over variables: even though WI and MI are highly correlated across possible worlds, as modelers we are free to set WI to the value we want without looking at the value of MI in the world within which we are intervening. However, an intervention on a variable can have downstream effects on variables that causally depend on it. For instance, in a possible world where Biden has won New York and has won the presidency with just 270 electoral college votes (i.e. NY=1, P=1), an intervention setting NY to 0 has the consequence of setting P to 0, since in the absence of the 29 electoral votes from NY Biden no longer has enough votes to win the presidency.

The **do operator** is an add-on to probability theory that enables us to reason about interventions. Specifically, $Pr(P|do(S))$ denotes the probability that P=1 given that we made an intervention setting S to 1.

We say that a state S was **necessary** for Biden to win the presidency in world W if in W we have S=1 (Biden wins the state), P=1 (Biden wins the

presidency), and an intervention setting S=0 would set P=0. In other words, a state is necessary if, had Biden not won the state (holding everything else about W constant), he would not have won the presidency.

Among the models described below, The Necessity-Sufficiency model, as well as the Counterfactual effect size model, rely on computations over possible worlds. Here we use two different sets of possible worlds: the simulations from FiveThirtyEight and from The Economist. Therefore, for each model of causal judgment that relies on computations over possible worlds, we computed separate sets of predictions for the version of that model calibrated with the simulations from FiveThirtyEight and for the version of that model calibrated with The Economist simulations.

1.2 Pivotality model

Chockler and Halpern (2004) introduce a simple measure of "graded responsibility". Formally, they define the degree of responsibility (d_r) of a variable C for an effect E as:

$$d_r = 1/(1 + k)$$

Where k is the number of variables other than C whose values would need to be changed by interventions in order to make C necessary for E. For instance, if in the actual world 3 committee members out of 5 voted in favor of a resolution, and the resolution needed a majority to be adopted, then each committee member who voted in favor has responsibility of $d_r = 1/(1 + 0) = 1$, since each member is 'already' necessary for the resolution to pass. By contrast, if all 5 committee members vote in favor, then a given committee member has $d_r = 1/(1 + 2) = 1/3$, since one would need to flip the vote of $k = 2$ other

committee members from "in favor" to "opposed" to make his vote necessary. In other words, a variable's degree of responsibility can be seen as a measure of 'how far away' the variable was from being pivotal to the outcome. There is some empirical evidence that distance from pivotality influences how people make attributions of responsibility (Lagnado, Gerstenberg & Zultan, 2013; Gerstenberg, Halpern & Tenenbaum, 2015).

Although framed as a model of responsibility judgments instead of a model of causal judgments, this model is a natural contender for a psychological model of causal judgments, because the notions of responsibility and causation are very closely related; indeed, some researchers consider the intuitive concepts of causation and responsibility to be essentially identical (Sytsma, Livengood & Rose, 2012). Moreso, this model explains some of the variance in attributions of responsibility in simple voting scenarios (Gerstenberg, Halpern & Tenenbaum, 2015) and is therefore appropriate for the present case study of causal judgments in the 2020 US presidential election.

The Pivotality model does not take the statistical properties of events into account, and therefore its predictions do not depend on a probability distribution over possible worlds. In the current context, this means that its predictions are independent of any forecasting model, and we only need to implement one version of the model. The degree of responsibility of a state for Joe Biden's victory can easily be computed analytically.

Define as a pivotal state a state such that, if we made that state go to Trump instead of Biden, while holding everything else constant, Biden would go from winning to not winning the presidency. Since Biden won 306 electoral votes, he has a margin of 36 electoral votes. The only blue state with more than 36 electoral votes is California (55), and therefore only California is a pivotal state in the actual world, with $d_r = 1$. But other states can be made

pivotal by first making interventions which make Trump win in other states.

Let B be the set of blue states (i.e. states won by Biden in the actual world). A state S can be made pivotal if we can find a subset b of B such that $S \notin b$, and:

$$36–EV(S) \leq \sum_b EV(b) < 36$$

Where $EV(S)$ is the number of electoral votes for state S, and $\sum_b EV(b)$ is the sum of electoral votes of states in b. If $\sum_b EV(b) > 36$, then making the states in b all go to Trump would be going too far, already making Biden lose without the contribution of S. If $\sum_b EV(b) < 36–EV(S)$, then making all the states in b go to Trump would not be enough to make S pivotal.

For all states with $EV(S) \geq 7$, then $b = \{NY\}$ makes S pivotal, since $EV(NY) = 29$, and we have

$36–7 \leq 29 < 36$

Therefore all these states have $k = 1$, and $d_r = 1/2$.

The remaining blue states all have $3 \leq EV(S) < 7$. States with between 4 and 6 EVs can be made pivotal by flipping (e.g) NY and VT ($29 + 3 = 32$ EVs). States with 3 EVs can be made pivotal by flipping (e.g.) PA and NJ ($20 + 14 = 34$ EVs). Therefore all these states have $k = 2$, and $d_r = 1/3$.

1.3 Necessity-Sufficiency model

Icard, Kominsky, and Knobe (2017) develop a model of actual causal strength that is based on two hypotheses about causal judgment:

(1) People make causal judgments by simulating different alternatives to what actually happened.

(2) People tend to consider a cause C as causing an effect E to the extent that C was necessary and robustly sufficient for E.

Formally, they define the causal strength of C for E as:

$$Pr(\neg C) \times Necessity(C \rightarrow E) + Pr(C) \times Sufficiency(C \rightarrow E)$$

Where $Pr(C)$ is the prior probability of C, and $Pr(\neg C) = 1 - Pr(C)$. $Necessity(C \rightarrow E)$ and $Sufficiency(C \rightarrow E)$ measure the "necessity strength" and "sufficiency strength" of C for E, respectively. Icard et al. (2017) give somewhat technical definitions of necessity and sufficiency strengths for the general case, so for concreteness we explain what they mean in the 2020 US presidential election case.

Let $S = 1$ if Biden wins state S, and $P = 1$ if Biden wins the presidency. Then $Necessity(S \rightarrow P)$ is simply a binary variable which takes the value 1 if winning state S was necessary in the actual world for Biden to win the presidency, and 0 otherwise.

Icard et al. (2017) remain agnostic about the exact mathematical definition of sufficiency strength, but they put forward two possibilities that are consistent with the experimental data that their model is designed to explain. The first measure is:

$$Sufficiency(S \rightarrow P) = Pr(P|do(S))$$

Where $Pr(P|do(S))$ denotes the probability of P given that we have performed an intervention setting S to 1 (Pearl, 2000). In other words, this measure defines sufficiency strength as the probability that Biden wins the election given that we intervened to make him win in state S. Icard et al. (2017) recognize that this measure is "particularly simple", and also suggest

a more plausible alternative. This alternative measure is:

$$Sufficiency(S \to P) = Pr(P|do(S), S = 0, P = 0)$$

In other words, the sufficiency strength of S for P is the probability that making Biden win in state S makes him win the presidency, given that he originally lost both S and the presidency. Algorithmically, we can compute this by selecting all the simulations where Biden lost both S and the presidency, and in each of these worlds, performing an intervention setting S to 1 and then recomputing whether Biden won the presidency. The sufficiency strength of S for P is simply the proportion of these worlds in which Biden wins the presidency after the intervention.

Of these two measures, the second is intuitively a better formalization of what it means for a cause to be 'sufficient' for an outcome. Therefore, when in the main text we refer to "the Necessity-Sufficiency model", we refer to the version that uses that second measure. However, for completeness we also computed and pre-registered causal ratings for the version of the model that uses the simpler first measure. Causal judgments from that version of the model were negatively correlated with human causal judgments (see section 3 below).

In sum, using the second measure of sufficiency strength, the Necessity-Sufficiency model defines the causal strength of S for P as:

$$Pr(\neg S) \times Necessity(S \to P) + Pr(S) \times Pr(P|do(S), S = 0, P = 0)$$

Algorithmically, we computed $Pr(S)$ by computing the proportion of simulations where Biden wins state S. Note that only California has a necessity strength of 1; other states have necessity strength of 0.

In total, we pre-registered causal judgments for 4 versions of the Necessity-Sufficiency model, one for each combination of sufficiency strength measure

(simple / complex) and forecasting model (FiveThirtyEight / The Economist). We report the results for the complex model in the main text, and the results for the simple model in section 2 below.

1.4 Counterfactual Effect Size Model

According to the Counterfactual effect size model (CESM; Quillien, 2020), the causal strength of C (cause) for E (effect) is a measure of the statistical effect size of C for E across possible worlds.

The intuition behind the model is that the causal strength of C for E is the average causal effect of C on E across possible worlds. The average causal effect is additionally standardized by the ratio of the standard deviations of C and E. The standardization ensures that the measure behaves as an effect size (so that one gets the same causal effect regardless of the unit of measurement; for example, this standardization would ensure that the causal effect of water intake on thirst would be the same regardless of whether we measure water volume in liters or gallons).

In the election case, the causal strength of state S for Biden's presidential victory (denoted as P) is computed in the following way:

a) Across all simulations, compute the standard deviation of S (denoted σ_S), the standard deviation of P (denoted σ_P), and the proportion of simulations where Biden wins state S (denoted $Pr(S)$).

b) For each simulation, create a 'twin simulation' by making an intervention setting S to a new, randomly sampled value. That is, make Biden win S in the twin simulation with probability $Pr(S)$ and lose with probability $Pr(\neg S)$. If the value of S is different between the simulation and its twin, compute the ratio of the change in the value of P between the two worlds to the change in

the value of S (denoted Δ_P / Δ_S).

c) Across all pairs of simulations compute the average value of Δ_P/Δ_S, then multiply this value by σ_S/σ_P. This is the causal strength of S for P.

Let us call this the 'full' version of the CESM. We also pre-registered causal judgments for a 'naïve' version of the model, where the causal strength of S for P is simply the correlation between S and P across possible worlds.

The motivation for this naïve model is the following. So far, existing data that support the CESM have come from studies where people were asked to reason about simple causal structures (e.g., Morris et al., 2019). In these simple causal structures, C and E typically obey the no-confounding condition (Pearl, 2000). The no-confounding condition states that $P(E|C) = P(E|do(C))$; in other words, the probability of E given that you observe C is equal to the probability of E given that you made C happen. In causal structures where C and E obey the no-confounding condition, the measure of causal strength in Quillien (2020) simply reduces to the correlation between C and E across possible worlds. Therefore, the empirical validity of the model has so far been tested by looking at whether people's judgments for a given causal structure were well-predicted by the correlation between C and E across possible worlds. Consequently, existing evidence is consistent with the possibility that, instead of computing causal strength in the way outlined in Quillien (2020), people use a simpler heuristic, computing the correlation between C and E even in causal structures that do not meet the no-confounding condition.

In the election case, the measure of causal strength given by the full version of the model does not reduce to the correlation between S and P across all simulations from a given forecasting model, because the no-confounding condition is not met. Both election forecasts assume that some states are correlated with each other (e.g., that if Biden wins Wisconsin he is disproportionately

also likely to win Michigan); this captures the fact that because of similarities between states (e.g., shared demographics), an election where Wisconsin and Michigan each go to different candidates is less likely than an election where they go for the same candidate. The no-confounding assumption is invalidated by this correlation across states, because whereas $P(E|do(C))$ does not depend on the correlation between C and other states, $P(E|C)$ does.

In total, we pre-registered causal judgments for 4 versions of the CESM, one for each combination of complexity (naïve/full) and forecasting model (FiveThirtyEight / The Economist). We report the results for the full model in the main text, and the results for the naïve model in section 3 below.

1.5 Paired permutation tests

We ran paired permutation tests to compare the fits of different models to the human data.

To compare two models (call them "model 1" and "model 2"), we first z-scored the human ratings as well as the predictions of each model. Then for each observation (i.e. for each causal rating of one state by one human participant), we computed an error score for each model by taking the squared error between the model prediction and the human rating. We then randomly swapped the error scores between each model (such that half of the error scores of model 1 were replaced with those of model 2, and vice-versa). We then computed the difference in the root mean squared error of these new shuffled "model 1" and "model 2".

We repeated this process ten thousand times, to approximate a distribution of differences. We then computed a p-value by computing the proportion of simulations where the magnitude of the difference in fit between the two models

is at least as large as the one we empirically observed.

2 Other models of causal cognition

Here we briefly discuss other formal theories of causal judgment, and why they do not apply to the present case, and/or why it is difficult to derive predictions from them.

2.1 Models of causal induction

Some formal theories of causal cognition aim at modeling the process of causal induction: how people infer the correct causal structure (including the strength of the causal relationships) underlying a given phenomenon (e.g., Cheng, 1997; Griffiths & Tenenbaum, 2005; Bramley, Dayan, Griffiths & Lagnado, 2017). In the election context, this would correspond to modeling the process by which someone who is unfamiliar with the US electoral system would learn its rules (e.g., the Electoral College) by observing the outcomes of several elections. By contrast, in the current study, we assume that people already possess the relevant knowledge about the US electoral system. Our primary interest here is not in how people learn the causal structure of the US electoral system, but in how the knowledge contained in this causal structure translates into judgments about the 'best' way to describe the outcome of a particular presidential election. In other words, the problem of causal judgment that we study here is a problem of description rather than a problem of inference. Theories of causal induction investigate the latter kind of problem and therefore are not relevant to the current study.

2.2 Process theories

According to process theories, the human sense of causation is based on the mental representation of "forces". Roughly, saying that C caused E means that C exerted a force which affected the value of E (Wolff, 2007). For instance, we say that "the blue billiard ball caused the red billiard ball to go into the hole" because the blue ball collided with the red ball, exerting sufficient force to send it into the hole.

Gerstenberg, Goodman, Lagnado, and Tenenbaum (2021) put forward a hybrid approach that combines insights from process and counterfactual theories. According to their model, our sense of causation is grounded in different types of counterfactual judgments. As emphasized by traditional counterfactual theories, we care about *whether* E would have happened in the absence of C. For instance, would the red ball have gone into the hole if the blue ball hadn't hit it? Additionally, and as emphasized by process theories, we care about *how* E happens, because the exact way that E happens depends on which forces contributed to the event. For instance, imagine that a green ball hits the red ball, sending it hurtling toward the hole at a 50 degrees angle, and that the blue ball then hits the red ball. As a result, the red ball changes course slightly, but still ends up in the hole (from a 20 degrees angle). Although the blue ball did not make a difference to whether the red ball entered the hole, it made a difference to *how* it did (i.e. from an angle of 20 instead of 50 degrees). According to the hybrid model, if C makes a difference to the manner in which E happens, it may be considered as a cause of E even if it does not influence whether E happens.

Both approaches (purely force-based and hybrid) are most naturally at home in modeling physical events (e.g., billiard ball collisions, the trajectory

of vehicles), because Newtonian mechanics provide an unambiguous definition of "force" in that context. For example, it is easy to define "*how* causation" by reference to different trajectories that an object could take to reach a place. By contrast, in a social context like the US presidential election, the notion of "force" is not formally defined. It is also unclear how to quantify how much winning a given state changes how the election is won.

2.3 Dependence theories

The two theories we discuss next are conceptually close to the current model, in that they both rely on the idea that causal strength is a matter of counterfactual dependence and is influenced by the prior probability of events.

Morris et al. (2018) introduce the SAMPLE measure, a measure of actual causal strength which is designed to select the variable on which an intervention is most likely to bring about the effect. The measure was designed for use in causal structures that obey the no-confounding assumption (i.e. where $P(E|C) = P(E|do(C))$. The election case does not obey this assumption (see section 1 above), so the model makes no predictions in this context. The SAMPLE measure also predicts that only factors that are necessary for an event will be considered as causal, which is implausible in the present case as it would predict that only California will be assigned a non-zero causal rating by participants, as only an intervention flipping CA from Biden to Trump would change the outcome of the presidential election.

Halpern and Hitchcock (2015) design a model of graded causal judgment that is based on the notion that some worlds are more 'normal' than others. To simplify, C is considered a cause of E only if there is a counterfactual world that is more normal than the actual world, and we can use that world to show

that C (possibly in conjunction with other variables) was necessary for E. For instance, to show that Pennsylvania was a cause of Biden's election, one would need to show that there exists an intervention on Pennsylvania (possibly along with interventions on other states) that leads to a world which is more normal than the actual world, but where Biden loses the presidency. Among causes that meet this criterion, the less normal causes have the highest causal strength.

Halpern and Hitchcock (2015) leave it open how exactly one can interpret the concept of 'normality'. In the current election case it seems plausible to quantify the normality of a variable, or that of a world, as its prior probability under a given election forecasting model. However, while this quantity is easy to compute for a single variable in the causal structure like that of Biden winning a given state, it is more challenging to compute the prior probability of a world, given the complexity of the multivariate distributions over possible worlds that are defined by the forecasting models. Another concern with that approach is that when normality is interpreted in a probabilistic sense, the Halpern-Hitchcock model makes predictions that widely diverge from human causal judgments in some simple causal structures (see Morris et al. 2019). Furthermore, in the current election case, the Halpern-Hitchcock model would judge most states as non-causal; for instance, it is unlikely that one would find a world that is more probable than the actual world and where Biden lost Pennsylvania along with some other blue states (given that for each state that Biden won, he had a more than 50% prior probability of winning that state).

2.4 Exploratory analyses with other measures of causal strength

In section 2.1 we say that mathematical measures of causal strength designed for problems such as causal induction are not in principle a good match for the current task. However, it is still a priori possible that these measures account for people's judgments here. For example, maybe when people make judgments of actual causation they use the same algorithms that they use for causal induction. Here, we show that this is not the case.

We want to emphasize that the analyses below are not meant to be criticisms of these measures, since the measures were designed to deal with different problems than the one we are interested here. Rather, we want to show that for the current problem (modeling judgments about the actual cause of a specific event), extrapolating from models of other aspects of causal cognition (e.g. causal inference) is not a successful approach.

Just like we did for the CESM and the Necessity-Sufficiency model, we use election forecasts to quantify the probabilities we use to derive causal judgments from these models. For example, we compute the probability $Pr(P|do(S))$ by performing an intervention making Biden win state S in every simulation, and counting the proportion of simulations where Biden now wins the presidency.

2.4.1 Delta-P

The Delta-P (ΔP) measure (Jenkins & Ward, 1967) is defined as:

$$\Delta P = Pr(E|C) - Pr(E|\neg C)$$

One can also consider a natural extension, $Pr(E|do(C)) - Pr(E|do(\neg C))$,

which conditions on interventions instead of observations.

Delta-P was introduced in the context of psychological work on judgments of contingency, but the basic idea has also been independently applied, under other names, to many other contexts. For example, it is the basis of some measures of voting power (Gelman, Katz, & Tuerlinckx, 2002). In fields like epidemiology and econometrics, some causal inference techniques aim at estimating the 'average causal effect' (see Schafer & Kang, 2008; Holland, 1986), which is equivalent to the version of ΔP that conditions on interventions, when E is a binary variable (Sprenger, 2018).

In the current context, the causal strength of state S in bringing a presidential victory P is defined as:

$$\Delta P(S) = Pr(P|S) - Pr(P|\neg S)$$

.

The judgments made by this measure are only modestly correlated with human judgments, r(24) = .47, p = .01 (FiveThirtyEight), and r(24) = .24, p = .25 (The Economist).

We also generated judgments for the version of ΔP which conditions on interventions, that is, $Pr(P|do(S)) - Pr(P|do(\neg S))$. The judgments made by this measure are only weakly correlated with human judgments, r(24) = .36, p = .07 (FiveThirtyEight), r(24) = .26, p = .20 (The Economist).

2.4.2 Probability of Necessity of Sufficiency (PNS)

Pearl (1999) introduces a measure of the Probability of Necessity and Sufficiency (PNS) of an event for an outcome. PNS is the probability that C is either necessary or sufficient for E. That is, it is the probability that, either C

and E hold and preventing C is enough to prevent E, or C and E do not hold and making C happen is enough to make E happen. Formally, in the present context it is defined as:

$$PNS = Pr(S, P)Pr(\neg P|do(\neg S), P, S) + Pr(\neg S, \neg P)Pr(P|do(S), \neg P, \neg S)$$

Here, PNS is equivalent to the version of ΔP which conditions on interventions. Therefore it is not a good match to human judgments.

Proof. To evaluate the probability of necessity (the first term of the PNS formula), we can simply perform an intervention setting S to 0 in every simulated election, and count the proportion of simulations in which P changes from 1 to 0. This is given by $Pr(P) - Pr(P|do(\neg S))$. Similarly, to evaluate the probability of sufficiency (the second term of the PNS formula), we can simply perform an intervention setting S to 1 in every simulated election, and count the proportion of simulations in which P changes from 0 to 1. This proportion is given by $Pr(P|do(S)) - Pr(P)$.

Thus we have:

$$PNS = Pr(P) - Pr(P|do(\neg S) + Pr(P|do(S)) - Pr(P)$$

$$= Pr(P|do(S)) - Pr(P|do(\neg S))$$

2.4.3 Power PC

Power-PC was a model of causal induction introduced by Cheng (1997). We note that Power-PC was meant to model causal inference in disjunctive causal structures, i.e., situations where an event C can on its own be sufficient to

cause E, independently of other causes. The election case is not a disjunctive causal structure, so there are no strong reasons to expect that Power-PC would be a good model of causal judgment in the current context, even if we were interested in people's causal induction abilities. Nevertheless, the measure is well-known, so we wanted to show that it could not account for the present results.

According to Power-PC, the causal strength of event C for outcome E is defined as:

$$P_c(C) = \frac{\Delta P}{Pr(\neg E|\neg C)}$$

where $\Delta P = Pr(E|C) - Pr(E|\neg C)$. In the current context, this translates to:

$$P_c(S) = \frac{\Delta P}{Pr(\neg P|\neg S)}$$

where $\Delta P = Pr(P|S) - Pr(P|\neg S)$

Causal strenght scores from Power-PC are moderately correlated with human judgments, r(24) = .59, p = .002 (FiveThirtyEight), r(24) = .58, p = .002 (The Economist).

3 'Simple' versions of the Necessity-Sufficiency model and CESM

As explained in section 1 above, in addition to the versions reported in the main text, we also tested 'simple' versions of the Necessity-Sufficiency model and of the CESM. We report their fit to the human data here.

The causal judgments from the simple version of the Necessity-Sufficiency model were negatively correlated with human judgments at r = -.60 (p = .001) for The Economist, and r = -.66 ($p < .001$) for FiveThirtyEight.

By contrast, the simple version of the CESM had a close fit to human causal judgments, with r = .74 ($p < .001$) for The Economist, and r = .80 ($p < .001$) for FiveThirtyEight; see figure S.1. In comparison, both versions of the full model have fits to the human data of r = .77. Thus, the current data do not seem diagnostic as to which version of the model (simple vs full) best accounts for people's causal judgments[1]. We note, however, that the non-linear nature of the relationship between the predictions of the full model and human judgments may make the Pearson's correlation coefficient an unreliable measure of model fit. In exploratory analyses, we used Spearman's rank-order correlations (which are less sensitive to the degree to which the dependent variable scales linearly with the independent variable), and found fits for the full model of ρ = .76 and $\rho = .83$ for The Economist and FiveThirtyEight, respectively. (For the simple model, the fits are $\rho = .67$ and $\rho = .50$, respectively).

[1]Note also that the full and simple versions make judgments that are highly correlated to each other (r = .76 for the version calibrated with the FiveThirtyEight data; r = .83 for the version calibrated with The Economist)

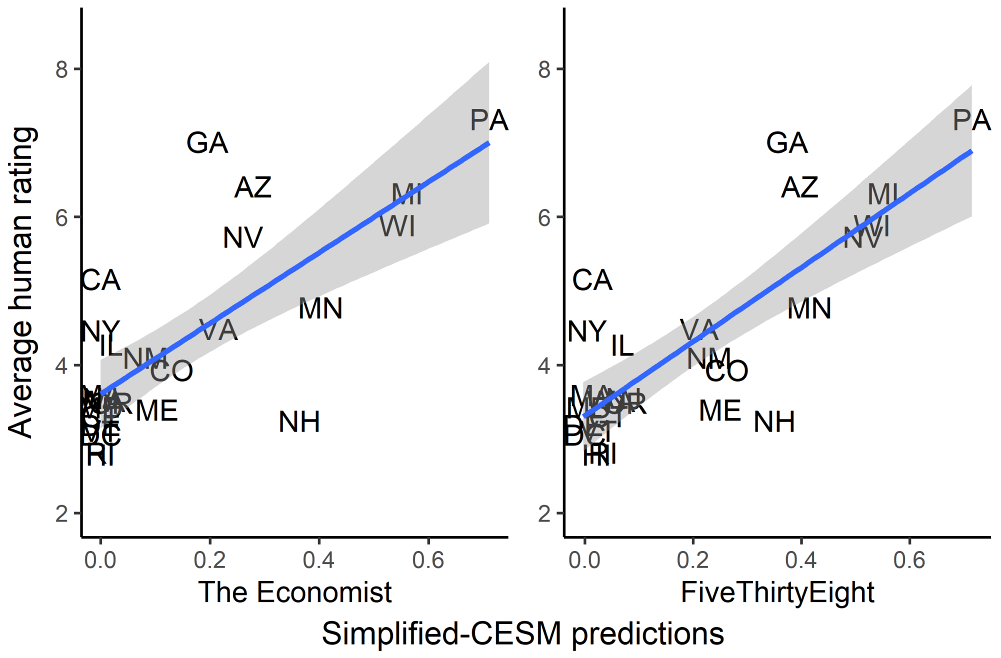

Figure S1: Correlation between the simplified CESM predictions and average human causal rating, across states.

4 Additional figures

4.1 Histograms of causal ratings, broken down by state

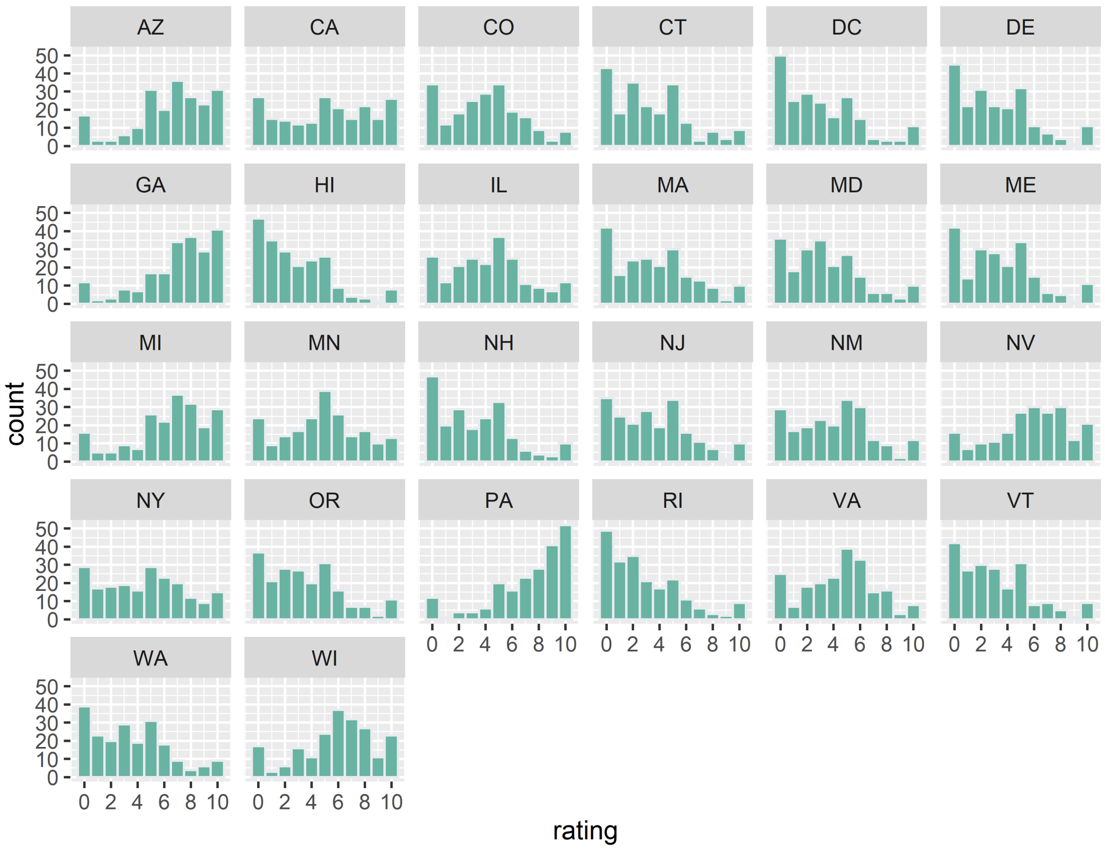

Figure S2: Frequency of causal ratings, broken down by state

5 Modified U.S. political knowledge quiz

The 5-question multiple-choice U.S. political knowledge quiz was modified from Delli Carpini and Keeter (1993). The questions are listed below. The questions and response options were displayed in randomized order.

(1) Who is the Vice President of the United States? [Michael Pence / Mike Pompeo / Mitch McConnell / Nancy Pelosi]

(2) Whose responsibility is it to determine if a law is constitutional or not? [The President / Congress / The Supreme Court / The Speaker of the House]

(3) How many electoral votes are required, at minimum, to win the presidency of the United States? [26 / 51 / 270 / 538]

(4) At present, which party has the most members in the House of Representatives in Washington, D.C.? [Democratic Party / Republican Party]

(5) Which party is more conservative at the national level? [Democratic Party / Republican Party]

References

Bramley, N. R., Dayan, P., Griffiths, T. L., & Lagnado, D. A. (2017). Formalizing Neurath's ship: Approximate algorithms for online causal learning. *Psychological review, 124*(3), 301.

Cheng, P. W. (1997). From covariation to causation: A causal power theory. *Psychological review, 104*(2), 367.

Chockler, H., & Halpern, J. Y. (2004). Responsibility and blame: A structural-model approach. *Journal of Artificial Intelligence Research, 22*, 93-115.

Delli Carpini, M. X., & Keeter, S. (1993). Measuring Political Knowledge: Putting First Things First. *American Journal of Political Science, 37* (4), 1179-1206. https://doi.org/10.2307/2111549.

Gelman, A., Katz, J., & Tuerlinckx, F. (2002). The mathematics and statistics of voting power. *Statistical Science, 17*(4):420–435.

Gerstenberg, T., Halpern, J. Y., & Tenenbaum, J. B. (2015). Responsibility judgments in voting scenarios. *Proceedings of the cognitive science society.*

Gerstenberg, T., Goodman, N. D., Lagnado, D. A., & Tenenbaum, J. B. (2021). A counterfactual simulation model of causal judgments for physical events. *Psychological Review (forthcoming)*

Griffiths, T. L., & Tenenbaum, J. B. (2005). Structure and strength in causal induction. *Cognitive psychology, 51*(4), 334-384.

Halpern, J. Y., & Hitchcock, C. (2015). Graded causation and defaults. *The British Journal for the Philosophy of Science, 66*(2), 413-457.

Halpern, J. Y. (2016). *Actual causality.* MIT Press.

Heidemanns, M., Gelman, A., & Morris, G. E. (2020). An Updated Dynamic Bayesian Forecasting Model for the US Presidential Election. *Harvard Data Science Review, 2*(4). https://doi.org/10.1162/99608f92.fc62f1e1

Holland, P. W. (1986). Statistics and Causal Inference. *Journal of the American Statistical Association, 81*:945–960.

Icard, T. F., Kominsky, J. F., & Knobe, J. (2017). Normality and actual causal strength. *Cognition, 161*, 80-93.

Jenkins, H. M. & Ward, W. C. (1965). Judgment of contingency between responses and outcomes. *Psychological Monographs: General and Applied, 79*(1):1–17.

Morris, A., Phillips, J. S., Icard, T., Knobe, J., Gerstenberg, T., & Cushman, F. (2018). Causal judgments approximate the effectiveness of future interventions. *PsyArXiv*

Morris, A., Phillips, J., Gerstenberg, T., & Cushman, F. (2019). Quantitative causal selection patterns in token causation. *PLoS one, 14*(8), e0219704.

Lagnado, D. A., Gerstenberg, T., & Zultan, R. I. (2013). Causal responsibility and counterfactuals. *Cognitive science, 37*(6), 1036-1073.

Pearl, J. (1999). Probabilities of causation: three counterfactual interpretations and their identification. *Synthese, 121*(1-2):93–149.

Pearl, J. (2000). *Causality.* Cambridge university press.

Quillien, T. (2020). When do we think that X caused Y?. *Cognition, 205*, 104410.

Schafer, J. L. and Kang, J. (2008). Average causal effects from nonrandomized studies: a practical guide and simulated example. *Psychological methods, 13*(4):279.

Silver, N. (2020, August 12). *How FiveThirtyEight's 2020 Presidential Forecast Works — And What's Different Because Of COVID-19.* FiveThirtyEight. https://fivethirtyeight.com/features/how-fivethirtyeights-2020-presidential-forecast-works-and-whats-different-because-of-covid-19/ Last retrieved 01/06/2021

Sprenger, J. (2018). Foundations of a probabilistic theory of causal strength. *Philosophical Review, 127*(3), 371-398.

Sytsma, J., Livengood, J., & Rose, D. (2012). Two types of typicality: Rethinking the role of statistical typicality in ordinary causal attributions. *Studies in History and Philosophy of Science Part C: Studies in History and Philosophy of Biological and Biomedical Sciences, 43*(4), 814-820.

Wolff, P. (2007). Representing causation. *Journal of experimental psychology: General, 136*(1), 82.
